# Understanding carbon regulation in aquatic systems - Bacteriophages as a model

**DOI:** 10.12688/f1000research.6031.1

**Published:** 2015-06-01

**Authors:** Swapnil Sanmukh, Krishna Khairnar, Waman Paunikar, Satish Lokhande

**Affiliations:** 1 Environmental Virology Cell, National Environmental Engineering Research Institute (NEERI), Nagpur, Maharashtra, 440020, India; 2 Analytical Instrumentation Division (AID), CSIR-NEERI, Nehru Marg, Maharashtra, Nagpur-440020, India

**Keywords:** interconversion, microbial carbon pump, carbon sequestration, refractory carbon, global carbon cycle

## Abstract

The bacteria and their phages are the most abundant constituents of the aquatic environment, and so represent an ideal model for studying carbon regulation in an aquatic system. The microbe-mediated interconversion of bioavailable organic carbon (OC) into dissolved organic carbon (DOC) by the microbial carbon pump (MCP) has been suggested to have the potential to revolutionize our view of carbon sequestration. It is estimated that DOC is the largest pool of organic matter in the ocean and, though a major component of the global carbon cycle, its source is not yet well understood. A key element of the carbon cycle is the microbial conversion of DOC into inedible forms. The primary aim of this study is to understand the phage conversion from organic to inorganic carbon during phage-host interactions.

Time studies of phage-host interactions under controlled conditions reveal their impact on the total carbon content of the samples and their interconversion of organic and inorganic carbon compared to control samples. A total organic carbon (TOC) analysis showed an increase in inorganic carbon content by 15-25 percent in samples with bacteria and phage compared to samples with bacteria alone. Compared to control samples, the increase in inorganic carbon content was 60-70-fold in samples with bacteria and phage, and 50-55-fold for samples with bacteria alone. This study indicates the potential impact of phages in regulating the carbon cycle of aquatic systems.

## Introduction

The regulation of carbon in aquatic systems is a major biogeochemical process. The oceans’ surface takes up about 2% more CO _2_ gas than they release, a proportion of which dissolves into the water, forming carbonic acid. The increase in CO _2_ levels in oceans decreases the pH, resulting in acidification which affects the oceanic ecosystem ^1^. Carbon also enters the seas through the food web via photosynthesis, but does not last for long periods and is either released into the atmosphere as CO _2_ or sinks to the ocean depths as dead organic matter. However, a significant amount of carbon is present in the water in the form of DOC ^2, 4, 5^. The roles that ocean viruses play are very important in shaping microbial population sizes as well as in regenerating carbon and other nutrients ^6– 8^. It is estimated that every second, approximately 10 ^23^ viral infections occur in the ocean. Therefore, it should not be surprising that viruses are major influential forces behind biogeochemical cycles ^5– 8^.

A key element of the carbon cycle is the microbial conversion of dissolved organic carbon into inedible forms. Microbes play a dominant role in “pumping” bioavailable carbon into a pool of relatively inert compounds. The microbial carbon pump (MCP) “may act as one of the conveyor belts that transports and stores carbon in oceans.” The MCP also appears to function in deep waters, where bacteria adapted to the high-pressure environment may be able to degrade refractory DOC. Hiroshi Ogawa *et al.*, showed that marine microbes are able to convert bioavailable DOC to refractory DOC ^2, 4, 5^.

The present communication represents time studies of phage-host interactions under controlled conditions, in order to analyze their impact on the total carbon content of the source (nutrient broth) and their interconversion between organic and inorganic forms of carbon with respect to control samples. The control sample is just the nutrient broth without the inoculation of bacterium and their respective phage.

## Materials and methods

The experiment was designed to measure the inorganic carbon levels in three conditions: control (nutrient broth only), bacteria alone and bacteria with their specific phage. The bacterium used during our study was *E. coli* (ATCC, strain 13706) and the bacteriophage used was phi X174 (ATCC, strain 13706 B1). They represent a good model for carbon conversion and interconversion through phage-host interactions and their interaction can be easily determined by the instruments like TOC analyzer ^3, 6, 7^.

All three experimental conditions were conducted in 1L of sterilized nutrient broth each as to have a defined composition of the nutrients available for our study (HiMedia Pvt. Ltd.). For the bacteria without phage condition, sterilized nutrient broth media was inoculated with 100 cfu/ml of *E. coli* (ATCC 13706) previously enriched and incubated at 37°C; for the bacteria with phage condition approximately 1 ml of 1000 pfu/ml of phage were added. All flasks were sealed and incubated at 37°C for 18 hours. For control condition, sterile uninoculated nutrient broth was kept at 4°C throughout the experiment.

The initial reading were analyzed by a total organic carbon (TOC) analyzer (Shimadzu, Japan Model: TOC-Vcph) after 18 hours of incubation for all three sets of samples were recorded as “0” hours reading and before inoculation of bacteria and phages (see Table 1 and Table 2). TOC analysis was further carried out after every 2 hours until a stationary state was achieved. The stationary phase for inorganic carbon was defined by no further increase or decrease in the reading of inorganic carbon.

**Table 1.** TOC analysis results of control and bacterial samples (with and without phage).

Experiment No. 1	Control 1 (ppm)			Sample without phage 1 (ppm)			Sample with phage 1 (ppm)		
Time (hours)	TOC	TC	IC	TOC	TC	IC	TOC	TC	IC
**0**	2915	2916	0.7118	2740	2769	28.91	2780	2811	31.53
**2**	2834	2834	0.9182	2818	2847	28.91	2788	2818	29.72
**4**	2507	2508	0.9432	2162	2193	29.86	2209	2239	31.38
**6**	2436	2437	0.8439	2301	2327	24.77	2517	2543	25.34
**8**	2152	2153	1.064	1921	1946	22.27	1906	1929	25.89
**10**	1929	1930	0.8917	1530	1562	22.24	1372	1394	31.51
**12**	1887	1888	0.9637	1757	1798	31.27	1496	1528	31.93
**14**	1827	1828	0.9217	1415	1458	43.09	1759	1809	50.66
**16**	1903	1957	0.9926	1658	1787	55.47	1844	2050	66.94
**18**	2169	2259	1.0459	1931	2043	63.19	2078	2279	74.41
**20**	2391	2438	1.0937	2179	2305	79.54	2367	2399	89.23
**22**	2613	2695	1.1853	2444	2517	87.92	2574	2583	102.11
**24**	2880	2882	1.238	2689	2784	94.76	2648	2764	116.4
**26**	2741	2742	1.751	2726	2811	85.83	2684	2789	105.5
**28**	3333	3332	1.557	3047	3126	79.59	3091	3196	105.5

**Table 2.** TOC analysis results of control and bacterial samples (with and without phage).

Experiment No. 2	Control 2 (ppm)			Sample without phage 2 (ppm)			Sample with phage 2 (ppm)		
Time (hours)	TOC	TC	IC	TOC	TC	IC	TOC	TC	IC
**0**	3041	3042	0.7992	2789	2818	28.96	2844	2871	27.47
**2**	2871	2872	0.9459	2922	2951	28.61	2756	2794	37.72
**4**	2573	2574	0.8808	2360	2389	29.13	2365	2396	31.26
**6**	2167	2168	0.8449	2345	2370	24.77	2286	2319	33.11
**8**	2184	2185	1.039	1935	1957	23.16	1953	1983	30.04
**10**	1456	1457	1.004	1574	1600	25.94	1536	1570	33.44
**12**	1907	1908	0.9637	1819	1852	34.15	1592	1630	37.37
**14**	1631	1632	0.9014	2032	2115	64.52	2023	2088	82.56
**16**	1875	1917	1.0013	2197	2283	73.79	2113	2193	90.15
**18**	2047	2132	1.1021	2367	2378	86.21	2281	2284	97.58
**20**	2294	2353	1.2008	2429	2541	92.34	2335	2409	104.91
**22**	2455	2506	1.3502	2609	2766	97.88	2449	2523	111.63
**24**	2679	2681	1.421	2752	2853	100.9	2538	2657	119
**26**	2773	2775	1.533	2779	2877	98.77	2701	2818	116.8
**28**	3244	3245	1.65	3157	3250	92.22	3005	3113	107.2

**Figure 1.**
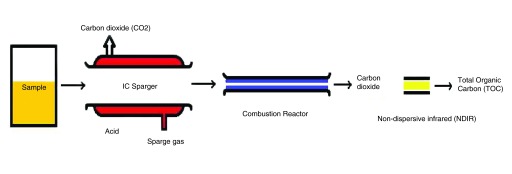
Principle of TOC analysis.

**Figure 2.**
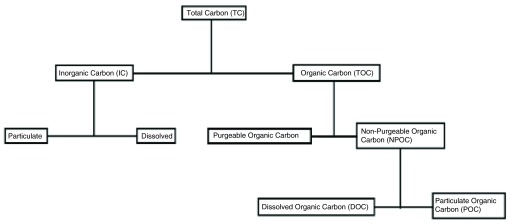
Flow chart showing ingredient components of total carbon.

Please refer Figure 1 and Figure 2 for understanding the principle of TOC analysis and different types of carbon compounds. The overall experiment was repeated for 10 times and their averages are represented in the Table 1 and Table 2.

**Figure 3.**
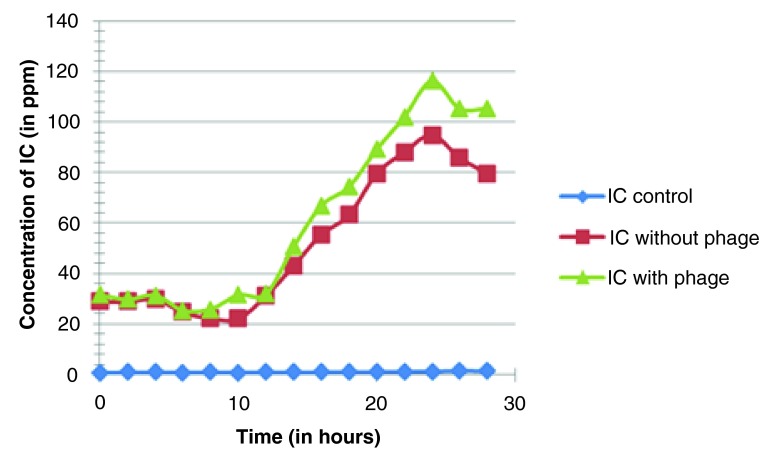
Variation in inorganic carbon content (in ppm) with respect to time (in hours).

**Figure 4.**
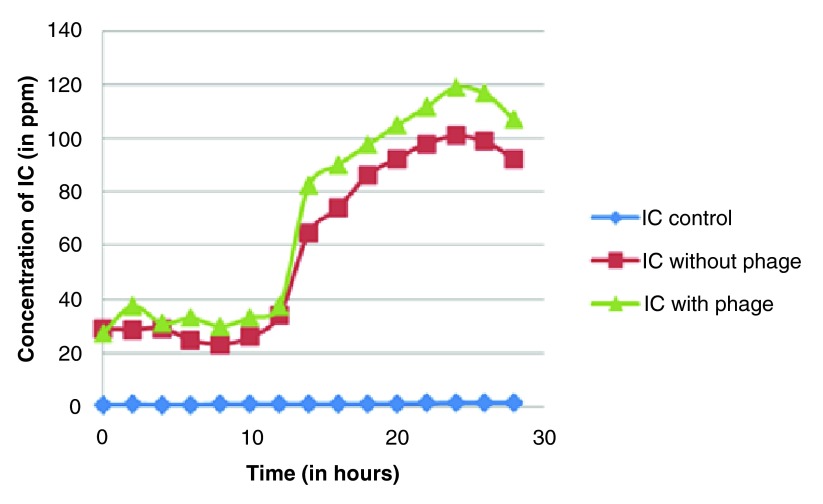
Variations in inorganic carbon content (in ppm) with respect to time (in hours).

## Results

The average results of the three sets are represented in Table 1 and Table 2, which show that the inorganic carbon content of the samples increased over time (except control) in both sets. The sample set with host-phage inoculation showed a increased reading of inorganic carbon levels compared to bacteria-only. There was an average 15–25 percent increase in inorganic carbon composition of sample set with host-phage inoculation. The result indicates that the phages may have role in regulation of carbon in aquatic systems through carbon sequestration or conversion in different biologically unavailable forms and can elevate inorganic carbon content levels in aqueous environments.

## Discussion

The increase in inorganic carbon content may be due to lysis of the host cell releasing its refractory carbon compounds and respiration produced CO _2_ during utilization of carbon constituent for phage assembly and development. These controlled experiment mimics the continuous viral infections occurring in the different aquatic environments ^2, 4, 5^. The consistent rise in the inorganic content is an indicator that, viruses somehow, seems to regulate carbon cycle to a greater extent as observed from the increase in IC level. The analytical results as indicated from the TOC analyzer are sole representation of phage lyses event and are worth analyzing further. If we are able to understand the biochemical mechanism and the byproducts generated during this whole process we may be able to determine the carbon sequestration in a better way. Considerable research activity needs to be initiated involving different environments conditions, parameters, sources, etc to facilitate better understanding of viral life cycle involving carbon cycle as an important area of future research. It can be proposed that carbon conversation during these studies gives us the clear ideas of the possible fate of carbon cycle and the role of phages. Similarly, we can also try to elucidate the role of phages (viruses) influencing other biogeochemical cycles including Nitrogen and Sulphur by using CHNS analyzer for better understanding of this process. It is also known that the infection of microbes also alters host metabolism significantly. Carbon sequestering algae like cyanobacteria are infected by cyanophages, which complicates our understanding further and demanding further in-depth studies. Lysogenic condition established by viruses under nutrient depleted condition or harsh environment can regulate the carbon utilization processes differently. Hence, the effect of viral infection on host metabolism remains unknown ^5– 8^.

Future work is essential for understanding the cellular processes especially infected (Lysogenic) host species. It will also prove helpful in deciphering the role of phages in regulating the carbon flow in the aquatic systems like oceans where their concentration outnumbered other species.
