# Supplementary material for: Understanding carbon regulation in aquatic systems - Bacteriophages as a model
Source: F1000Res. 2015 Jun 1;4:138. [Version 1] doi: 10.12688/f1000research.6031.1 (PMC4505785; doi:10.12688/f1000research.6031.1)
Supplement: Peer review history

# Peer review: Referee response for version 1

**DOI:** 10.5256/f1000research.6457.r9219

**Published:** 2015-07-15

**Authors:** Mayur Bharat Kurade

**Affiliations:** 1 Department of Natural Resources and Environmental Engineering, Hanyang University, Seoul, Korea, South

This paper deals with Bacteriophages as a model for carbon regulation in aquatic systems. The increase in inorganic carbon content was 60-70-fold in samples with bacteria and phage, and 50-55-fold for samples with bacteria alone being reported by the authors. The authors presented their experimental results quite well enough, that can be easily understandable to the readers. This manuscripts meets the necessary standards for this journal. The authors should pay attention on few of the concerns enlisted below. This manuscript should be acceptable after the changes suggested herewith.

 

1. The figures  (3 and 4) are not mentioned in the manuscript text, The discussion part may be elaborated to have much clear idea of the present work and its impact.
2. Please consider to use abbreviations instead of full forms, wherever necessary, e.g. '2 h' instead of '2 hours' in Materials and methods section, and '%' instead of 'percent' in "Results" section.
3. Please follow the reference styles with the journal guidelines. e.g. Hiroshi Ogawa *et al.*, ....should be corrected as Ogawa *et al*., followed by reference number in superscript. Reference number 3 is missing in introduction. References should be arranged in proper sequence. Please check all other references.
4. Authors should elaborate the 'Results section', as results of Fig. 1 to 4 are not discussed. I suggest author to overcome short details in this particular section.

I have read this submission. I believe that I have an appropriate level of expertise to confirm that it is of an acceptable scientific standard.

# Response

**Published:** 2015-07-15

**Authors:** Swapnil Sanmukh

**Affiliations:** CSIR, India

Dear Sir,

First of all thank you for approving our manuscript.

Regarding your queries:

-We will change the abbreviations for the units used in the figures and results.

-We will modify the reference style not properly cited for Hiroshi Ogawa *et al* as Ogawa *et al*.

-We will elaborate the discussion as well as result section and cite the figures which are not mentioned or not discussed. 

We would like to thanks for your time and suggestions for improving the quality of our manuscript.

# Peer review: Referee response for version 1

**DOI:** 10.5256/f1000research.6457.r8840

**Published:** 2015-06-22

**Authors:** Balendu Shekher Giri

**Affiliations:** 1 Centre for Biofuels and Biotechnology Division, Council of Scientific and Industrial Research (CSIR), Thiruvananthapuram, India

This paper deals with an understanding of the use of Bacteriophages as a model for carbon regulation in aquatic systems The results show the increase in inorganic carbon content by 15-25 percent in samples with bacteria and phage compared to samples with bacteria alone with comparing to control samples, the increase in inorganic carbon content was 60-70-fold in samples with bacteria and phage, and 50-55-fold for samples with bacteria alone being reported by the authors. The biogeochemical process of carbon in aquatic environment is well discussed in the manuscript. The manuscript has been written in good English and journal guidelines have been followed strictly. The paper should be indexed only after revision by the authors.

1. Abstract: This section is written very well with all the results and basic concepts. This section is also explaining the present work done by the authors. 
2. Introduction, Page 2, Line 3: "The oceans’ surface takes up about 2% more CO _2_  gas than they release, a proportion of which dissolves into the water, forming carbonic acid”  requires a reference. 
3. Introduction, Page 2, Line 6: “Carbon also enters the “seas” through the food web via photosynthesis, but does not last for long periods and is either released into the atmosphere as CO _2_ or sinks to the ocean depths as dead organic matter”. The “seas” should be “sea” and a reference is required.
4. Introduction, Page 2, Line 23: Reference “Hiroshi Ogawa *et al*.,” should come with the year of publication. 
5. Material and methods, this section is written very well and you can understand the experiments conducted by the authors. I have only some queries and suggestions for this section:
  Page 2, Line 8: Kindly provide the manufacturer information for the TOC analyzer in the first usage.
6. Result section is very short (even shorter than abstract) but it’s very informative and well written.
7. Discussion section is also very well written with comparative study of this present work. But I think if you provide 2 or 3 more references then it will be better for this publication.
8. Conclusion; I think authors have been forgotten to provide the conclusion part of this work and it is very important. Authors should be adding this section.
9. Table 1 and 2 are presented very well and it’s looking like whole results are explained by them.
10. Figure 3 and 4 are not cited in the text and it should be cited. These figures are the main results and could be explained in the results and discussion section.
11. Figure 1 and 2 are presented very well and for figure 3 and 4 authors may modify with removing “in hours” by “h” and “in ppm” by “ppm”.
12. Figure 3 and 4: I am unable to understand what differences between both figures are because all the things are same. Should be for table 1 and 2 (ppm 1 and ppm 2 should be come in the figure titles)?

I have read this submission. I believe that I have an appropriate level of expertise to confirm that it is of an acceptable scientific standard.

# Response

**Published:** 2015-06-22

**Authors:** Swapnil Sanmukh

**Affiliations:** CSIR, India

Dear Sir,

First of all thank you for approving our manuscript.

Regarding your queries:

-I think most of them are not quite critical but we will cite figure 3 and figure 4 as they are not cited in the article.

-The figures are represented as per journals guidelines.

-We will provide the make of TOC analyser when it is represented firstly in the article.

-We have done the experiments in duplicate so that we can maintain homogeneity and have reproducible results minimizing manual errors. 

-The conclusion part is not included because the article is a research note, not a research article and it is not mandatory for this type of article.

We would like to thanks for your time and suggestions for improving the quality of our manuscript.
